# Trametenolic Acid Ameliorates the Progression of Diabetic Nephropathy in db/db Mice via Nrf2/HO-1 and NF-*κ*B-Mediated Pathways

**DOI:** 10.1155/2022/6151847

**Published:** 2022-08-30

**Authors:** Qi Duan, Lingling Tian, Jiabing Feng, Xinbo Ping, Lihong Li, Hasnaa Yaigoub, Rongshan Li, Yafeng Li

**Affiliations:** ^1^Shanxi Key Laboratory of Kidney Disease, Shanxi Provincial People's Hospital (Fifth Hospital) of Shanxi Medical University, Taiyuan, 030012 Shanxi, China; ^2^The Third Clinical College, Shanxi University of Chinese Medicine, Taiyuan, 030000 Shanxi, China; ^3^Laboratory Animal Center, Shanxi Provincial People's Hospital (Fifth Hospital) of Shanxi Medical University, Taiyuan, 030012 Shanxi, China; ^4^School of Life Sciences, Institutes of Biomedical Sciences, Shanxi University, Taiyuan 030006, China; ^5^Department of Nephrology, Shanxi Provincial People's Hospital (Fifth Hospital) of Shanxi Medical University, Taiyuan, 030012 Shanxi, China; ^6^Core Laboratory, Shanxi Provincial People's Hospital (Fifth Hospital) of Shanxi Medical University, Taiyuan, 030012 Shanxi, China; ^7^Academy of Microbial Ecology, Shanxi Medical University, Taiyuan, China

## Abstract

Diabetic nephropathy (DN) is a fatal complication of diabetes and the main cause of end-stage renal disease. Due to the suboptimal effects of current treatments, there is an urgent need to develop new therapeutic strategies for DN. Trametenolic acid (TA), a lanostane-type tetracyclic triterpenoid, is one of the main active ingredients extracted from the natural product *Inonotus obliquus*. Our study was aimed at clarifying the potential protective effects of TA on DN and its underlying mechanism. In this research, C57BLKS/db (db/db) mice were used as the spontaneous DN model, and TA (10 mg/kg/d) was intraperitoneally injected for 4 consecutive weeks. Ratio of right kidney weight/body weight was calculated, and the contents of serum creatinine (Scr), blood urea nitrogen (BUN), and urine albumin were detected. The activities of superoxide dismutase (SOD) and catalase (CAT) and the contents of reductive glutathione (GSH) and malondialdehyde (MDA) were measured. The histopathological changes of renal tissues were observed by hematoxylin and eosin (HE), periodic acid-Schiff (PAS), and Masson staining. The protein expressions of nuclear factor erythroid 2-related factor 2 (Nrf2), heme oxygenase-1 (HO-1), NAD(P)H:quinone oxidoreductase-1 (NQO-1), nuclear factor kappa B (NF-*κ*B), proinflammation cytokine tumor necrosis factor-*α* (TNF-*α*), interleukin-6 (IL-6), interleukin-1*β* (IL-1*β*), Nephrin, and Podocin were detected by western blot assay. Immunohistochemistry was utilized to detect expressions of collagen III (COL-III) and fibronectin (FN). Our results showed that TA administration significantly reduced the ratio of right kidney weight/body weight, BUN, Scr, and urine albumin levels and alleviated the histopathological changes of DN mice. Moreover, TA administration remarkably increased GSH content and SOD and CAT activities and decreased MDA content. Western blot assay demonstrated that TA activated Nrf2 signaling and increased the expression of downstream antioxidant enzymes HO-1 and NQO-1. Further studies illustrated that NF-*κ*B signaling was inhibited, and downstream proinflammation cytokine expressions of TNF-*α*, IL-6, and IL-1*β* were also downregulated. In addition, we also found that TA administration significantly increased the expression of nephrin and podocin proteins and reduced the protein expression of COL-III and FN. These findings suggested that TA exhibited a renoprotective effect by ameliorating oxidative stress and inflammation via Nrf2/HO-1 and NF-*κ*B signaling pathways.

## 1. Introduction

Diabetic nephropathy (DN), characterized by basement membrane thickening, glomerulosclerosis, tubulointerstitial fibrosis, and urinary albumin, is a fatal complication of diabetes and the main cause of end-stage renal disease (ESRD) [1]. Patients with DN are usually older, have a longer course of diabetes, and are often accompanied by comorbidities, so treatment strategies for DN are usually multifactorial [2, 3]. The current treatment of DN is mainly limited to the blockade of the renin-angiotensin-aldosterone system, and the therapeutic effect is not satisfying [4]. A crowed of people with DN develop to ESRD and enormous health care expenditures become a grievous socioeconomic burden on society [5]. Therefore, there is an urgent need to develop new therapeutic strategies for DN.

A number of studies have shown that the development of DN progression is related to renal damage caused by chronic inflammation, oxidative stress, fibrosis, and dyslipidemia [6, 7]. Nuclear factor erythroid 2-like 2 (Nrf2), a transcriptional factor that regulates oxidative stress reactions, is of high importance among crucial antioxidant defense proteins [8]. Under oxidative stress conditions, Nrf2 is activated and transfers to the nucleus, which upregulates the antioxidant defense enzyme heme oxygenase-1 (HO-1) and NAD(P)H:quinone oxidoreductase-1 (NQO-1) [9]. Nuclear factor kappa-B (NF-*κ*B) is a key intracellular molecule transcription factor in regulating the inflammatory response and mediates the expression of proinflammatory cytokines such as tumor necrosis factor-*α* (TNF-*α*), interleukin-1 *β* (IL-1*β*), and interleukin-6 (IL-6) [10]. Hence, targeting the Nrf2/HO-1 and NF-*κ*B signaling pathways may be a potential approach for the treatment of DN.

Natural herbs and their active ingredients have gradually become new therapeutic strategy for various diseases due to their high efficiency, small side effects, and low cost [9, 11, 12]. *Inonotus obliquus*, also known as chaga, is a kind of edible and medicinal fungus. It usually grows on birch trees, belonging to the Hymenochaetaceae family of Basidiomycetes [13]. *Inonotus obliquus* contains more than 200 active substances, among which polysaccharides, polyphenols, and triterpenes are the most abundant. Currently, *Inonotus obliquus* is mainly utilized to treat various cancers, diabetes, and heart diseases on account of its beneficial physiological functions such as lowering blood glucose, antioxidation, antitumor, and anti-inflammatory activities [13–18]. Trametenolic acid (TA) is the main component of lanostane-type tetracyclic triterpenoids [19]. TA was found to exhibit effective anticancer and anti-inflammatory activities in human prostatic carcinoma and breast cancer [20]. Moreover, TA had a scavenging effect on 2,2-diphenyl-1-picrylhydrazyl (DPPH) free radicals to some extent [21]. Nevertheless, there are few reports about the effect of TA on DN. Our research intends to investigate the effect of TA on DN mice and to delineate the underlying mechanism.

## 2. Materials and Methods

### 2.1. Animals and Groups

Twenty SPF 8-week-old male C57BLKS/db (db/db) mice and ten C57BL/6 mice were purchased from Guangdong Jinzhihe Biotechnology Co., Ltd. The db/db mice were randomly divided into two groups as follows: (1) DN group (*n* = 10) and (2) DN+TA group (*n* = 10). Mice in the DN+TA group were intraperitoneally injected with 10 mg/kg TA daily. The dose of TA was chosen according to the previous study [19]. C57BL/6 mice of the same age were considered as the normal control (NC) group (*n* = 10). The mice in the NC group and DN group were given normal saline for 4 consecutive weeks. All mice were housed at normal atmospheric temperature and relative humidity on a 12 h light/dark cycle. Animal procedures were performed in accordance with the National Institutes of Health (NIH) guidelines and were approved by the local animal care review committee.

### 2.2. Materials and Reagents

Nephrin antibody was procured from Abcam (Cambridge, MA. USA). Podocin antibody was bought from Invitrogen (Carlsbad, CA, USA). Nrf2, HO-1, NOQ1, NF-*κ*B, P-NF-*κ*B, TNF-*α*, IL-1*β*, IL-6, GAPDH, horseradish peroxidase- (HRP-) conjugated goat anti-mouse IgG, and goat anti-rabbit IgG antibodies were purchased from Cell Signaling Technology Inc. (Danvers, MA, USA). Urine albumin, serum creatinine (Scr), blood urea nitrogen (BUN), superoxide dismutase (SOD), catalase (CAT), reductive glutathione (GSH), and malondialdehyde (MDA) detection kits were purchased from Nanjing Jiancheng Bioengineering Institute (Nanjing, Jiangsu, China). Bicinchoninic acid (BCA) kit was procured from Beyotime Biotech (Shanghai, China). Chemiluminescent (ECL) reagent kit was purchased from Millipore (USA).

### 2.3. Extraction of TA

After drying to constant weight, *Inonotus obliquus* were crushed into powder with a particle size of 40 meshes and then soaked in 50% isopropyl alcohol for 0.5 h before being loaded into the chromatography column. After eluting with 50% isopropyl alcohol for 1 h, the extract was concentrated under reduced pressure at 50°C with 1/2 volume of the eluent. The upper ethyl acetate phase was collected after extracting with ethyl acetate for three times for 30 minutes. The dried powder of ethyl acetate was recovered under reduced pressure at 60°C and loaded into a C18 modified silica gel packed chromatography column then eluted with a gradient alcohol solution for 1 h. The 100% ethanol elution peak was recovered under reduced pressure at 60°C, and then the concentrated solution was freeze-dried at -80°C to obtain TA freeze-dried powder. Uniform suspension was prepared in the experiment.

### 2.4. Assessment of Kidney Function Parameters

Body weight and right kidney weight were assessed, and the ratio of right kidney weight/body weight was calculated. 24 h urine was collected one day before the end of the experiment. Samples of blood were centrifuged for 10 min, and then, the obtained serums were stored at -80°C for biochemical assays. The contents of blood urea nitrogen (BUN), serum creatinine (Scr), and urine albumin were estimated by kits according to the manufacturer's protocol.

### 2.5. Detection of Oxidative Stress Indicators

Serum levels of GSH and MDA and activity of SOD and CAT were detected according to the protocol provided in the reagent kits.

### 2.6. Histopathological Changes

Kidney tissues were fixed with 4% paraformaldehyde, embedded in paraffin, and cut into 3 *μ*m thick sections. The sections were stained with hematoxylin and eosin (HE), periodic acid-Schiff (PAS), and Masson staining. Twenty visual fields were randomly selected to observe the pathological changes of the kidneys. The images were analyzed using Image-Pro Plus 6.0 software to assess the degree of glomerular sclerosis and tubulointerstitial damage.

### 2.7. Immunohistochemical Analysis

The slides were washed with Tris-buffered saline (TBS) solution following deparaffinization and hydration and then soaked in methanol solution containing 3% H_2_O_2_ for 10 min. The slides were incubated with primary antibodies collagen III (COL-III) and fibronectin (FN) at 4°C overnight and then washed with TBS. The horseradish peroxidase-conjugated secondary antibody was added and reacted at room temperature for 1 h. An isotype-matched control antibody was used as a negative control. Twenty fields were randomly selected, and the relative expressions of COL-III and FN proteins were measured with Image-Pro Plus 6.0 software.

### 2.8. Western Blot Analysis

Protein samples were extracted by employing RIPA solution supplemented with the protease inhibitor PMSF. BCA protein quantitative assay kit was utilized to detect the concentration of protein samples. Equal amounts of protein were separated by SDS-PAGE, wet-transformed, and blocked with 5% nonfat milk for 1 h. Membranes were incubated at 4°C overnight with the following primary antibodies: Nrf2 (1 : 1000), HO-1 (1 : 1000), NQO-1 (1 : 1000), NF-*κ*B (1 : 1000), P-NF-*κ*B (1 : 1000), Nephrin (1 : 1000), Podocin (1 : 1000), TNF-*α* (1 : 1000), IL-1*β* (1 : 1000), IL-6 (1 : 1000), and GAPDH (1 : 1000). Then, the membranes were washed with TBST and incubated for 1 h at room temperature with horseradish peroxidase- (HRP-) conjugated secondary antibodies. Finally, the membranes were washed with TBST and exposed to ECL solution. Images were taken with the automatic gel imaging system (Bio-Rad ChemiDoc MP). Image-Pro Plus 6.0 software was employed for gray value analysis.

### 2.9. Statistical Analysis

All experiments were performed at least three times. Data were presented as mean ± standard deviation (SD) and analyzed by using SPSS 19.0 software. Differences between the groups were measured by one-way analysis of variance. A value of *P* < 0.05 was considered statistically significant.

## 3. Results

### 3.1. TA Administration Significantly Ameliorated Renal Function

**Figure 1:**
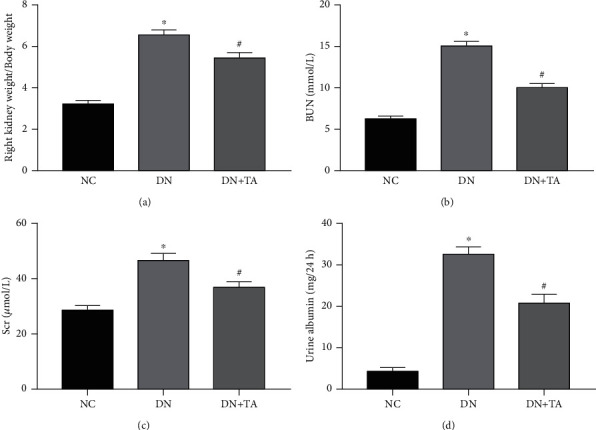
TA administration significantly ameliorated renal function in DN. Increased ratios of right kidney weight/body weight (a), BUN (b), Scr (c), and urine albumin (d) in DN mice were revised by TA. Data were expressed as mean ± SD (*n* = 10). ^∗^*P* < 0.05 versus NC group; ^#^*P* < 0.05 versus DN group.

In this research, db/db mice were used as the spontaneous DN model. After collecting urine, serum, and kidney samples, the ratio of right kidney weight/body weight, BUN, Scr, and urine albumin levels were detected. As shown in Figure 1, mice in the DN group exhibited an augmented ratio of right kidney weight/body weight, BUN, Scr, and urine albumin levels (*P* < 0.05) when compared with mice in the NC group, whereas TA administration significantly reduced the ratio of right kidney weight/body weight and the contents of BUN, Scr, and urine albumin (*P* < 0.05). All these data indicated that TA administration significantly ameliorated renal function in DN.

### 3.2. TA Administration Reduced Kidney Histological Alterations in DN

**Figure 2:**
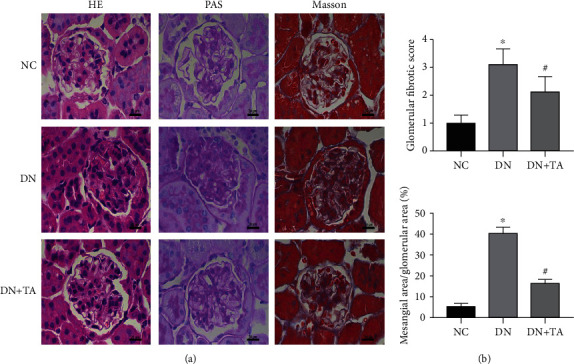
TA alleviated the renal histological alterations in DN. (a) Representative micrographs of HE, PAS, and Masson staining. Original magnification: 400x. (b) Quantitative analyses of mesangial area and Masson staining of glomerular. Data were expressed as mean ± SD (*n* = 10). ^∗^*P* < 0.05 versus NC group; ^#^*P* < 0.05 versus DN group.

After harvesting the kidney tissues, HE, PAS, and Masson staining were performed to assess the histological alteration. As shown in Figure 2, glomerular mesangial matrix expansion and collagen deposition were substantially augmented in the DN group when compared with the NC group (*P* < 0.05). Following TA treatment, glomerular mesangial matrix expansion and collagen deposition were substantially reduced (*P* < 0.05). These results confirmed that TA alleviated diabetic renal damage in DN mice.

### 3.3. TA Administration Mitigated Oxidative Stress in DN

**Figure 3:**
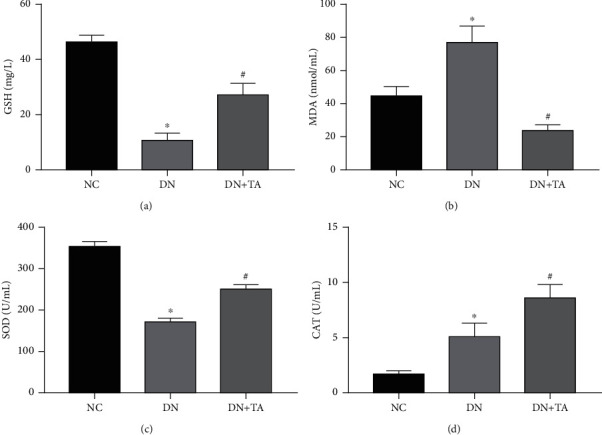
TA mitigated oxidative stress in DN. The decreased GSH (a) content, SOD (c), CAT (d) activities, and increased MDA (b) content were remarkably reversed by TA treatment. Data were expressed as mean ± SD (*n* = 10). ^∗^*P* < 0.05 versus NC group; ^#^*P* < 0.05 versus DN group.

To explore the effect of TA on oxidative stress in DN, we detected the contents of GSH and MDA and the activities of SOD and CAT. In comparison with the NC group, SOD activity was significantly decreased and the MDA content was significantly increased in DN mice (*P* < 0.05), which indicated that oxidative damage occurred owing to the increased levels of lipid peroxides in DN mice, while the content of GSH and the activity of CAT were extremely decreased. The decrease in GSH level and SOD and CAT activities and the increase in MDA content (*P* < 0.05) were remarkably reversed by TA treatment. Collectively, these data hinted that TA significantly reduced the lipid peroxide levels in vivo and augmented the antioxidant capacity of mice (Figure 3).

### 3.4. TA Administration Alleviated Oxidative Stress by Activating Nrf2/HO-1 Pathway in DN

**Figure 4:**
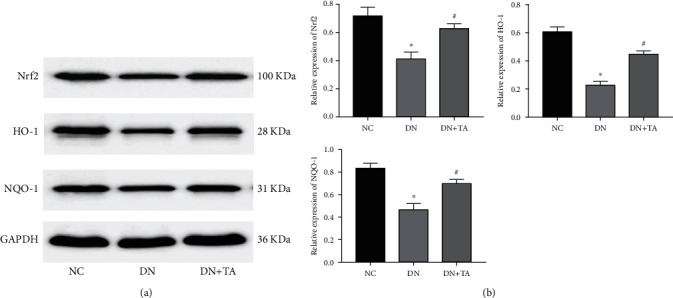
TA administration alleviated oxidative stress by activating Nrf2/HO-1 pathway in DN. (a) Representative western blots for Nrf2, HO-1, and NQO-1 protein expressions. (b) The quantification of Nrf2, HO-1, and NQO-1 protein expressions. All data are presented as mean ± SD. ^∗^*P* < 0.05 versus NC group; ^#^*P <*0.05 versus DN group.

To further investigate the mechanism of TA by which it alleviates oxidative stress in DN, the key proteins of Nrf2/HO-1 pathway were detected by western blot assay. As shown in Figure 4, the expressions of Nrf2, HO-1, and NQO-1 proteins were significantly decreased in the DN group compared with the NC group (*P* < 0.05). In contrast, treatment with TA dramatically increased the expressions of Nrf2, HO-1, and NQO-1 proteins (*P* < 0.05). All the results above proved that TA alleviated oxidative stress by activating Nrf2/HO-1 signaling pathway in DN.

### 3.5. TA Administration Attenuated Inflammatory Response through Inhibiting NF-*κ*B Signaling in DN

**Figure 5:**
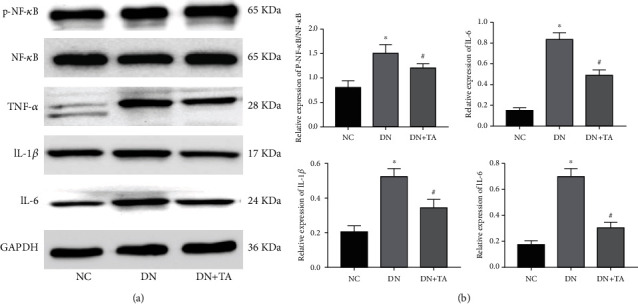
TA administration attenuated the inflammatory response through inhibiting NF-*κ*B signaling in DN. (a) Representative western blots for NF-*κ*B, P-NF-*κ*B, TNF-*α*, IL-1*β*, and IL-6 protein expressions. (b) The quantification of P-NF-*κ*B/NF-*κ*B, TNF-*α*, IL-1*β*, and IL-6 protein expressions. All data are presented as mean ± SD. ^∗^*P* < 0.05 versus NC group; ^#^*P* < 0.05 versus DN group.

To explore the effects of TA on the inflammatory response in DN and its underlying mechanism, the protein expressions of NF-*κ*B and P-NF-*κ*B and downstream proinflammatory factors TNF-*α*, IL-1*β*, and IL-6 were measured by western blot assay. As illustrated in Figure 5, TA administration dramatically decreased the protein expression of P-NF-*κ*B/NF-*κ*B and proinflammatory factors TNF-*α*, IL-1*β*, and IL-6 (*P* < 0.05). Our data indicated that NF-*κ*B signaling was involved in the inflammatory response in DN progression.

### 3.6. TA Administration Showed a Protective Effect on Podocyte Damage in DN

**Figure 6:**
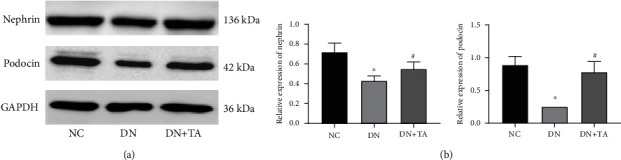
TA administration exhibited a protective effect on podocyte damage in DN. (a) Representative western blots for Nephrin and Podocin protein expressions. (b) Quantification of Nephrin and Podocin protein expressions. All data are presented as mean ± SD. ^∗^*P* < 0.05 versus NC group; ^#^*P* < 0.05 versus DN group.

Accumulating evidences indicate that podocytes are vital for the renal glomerular filtration barriers, which play an important role in the pathogenesis of DN. As shown in Figure 6, compared with the NC group, the protein expression levels of Nephrin and Podocin in the DN group were significantly downregulated (*P* < 0.05). After treatment with TA, Nephrin and Podocin expression levels were dramatically boosted (*P* < 0.05). The data above revealed that the reduction of nephrin and podocin expression in DN indicated a direct toxic effect on podocytes, while this effect was reversed by TA.

### 3.7. TA Administration Reduced Renal Fibrosis in DN

**Figure 7:**
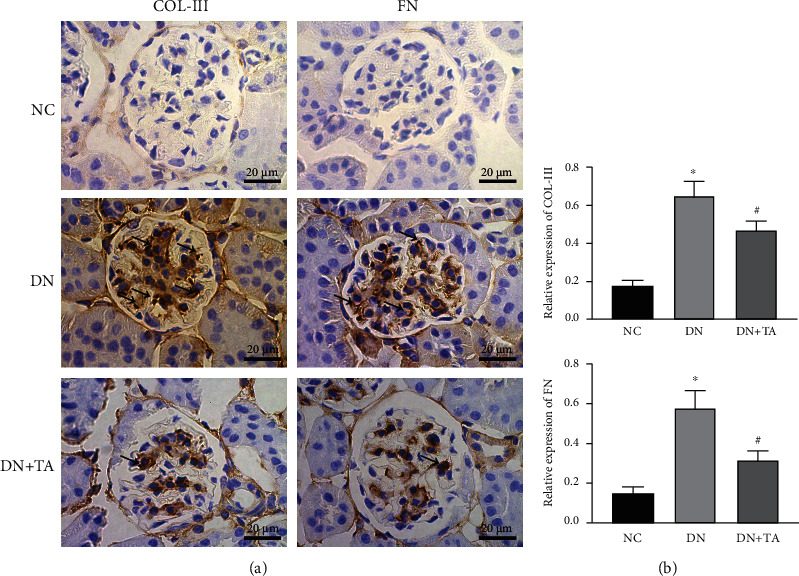
TA administration reduced renal fibrosis in DN. (a) Immunohistochemistry of COL-III and FN. Original magnification 400x. (b) Semiquantitative analyzation of COL-III and FN expressions. The arrows in the kidney section indicate that COL-III and FN are highly expressed areas. Data were expressed as mean ± SD (*n* = 10). ^∗^*P* < 0.05 versus NC group; ^#^*P* < 0.05 versus DN group.

To investigate the effects of TA on renal fibrosis, the expression levels of COL-III and FN were detected by immunohistochemistry. The levels of COL-III and FN in DN group were markedly higher than those in NC group (*P* < 0.05). After TA administration, the expression levels of COL-III and FN were significantly decreased (*P* < 0.05) (Figure 7). These findings suggested that TA repressed the expressions of COL-III and FN and exhibited an antifibrotic effect on diabetic renal injury.

## 4. Discussion

DN, a leading cause of ESRD, causes serious health lesions and induces a great financial burden to human society. So far, there is no effective treatment strategy for DN [22]. Increasing researches have been conducted to probe the effects and mechanisms of natural herbs and their bioactive ingredients in treating and managing DN [9]. TA is the main active component of triterpenoids, which has outstanding protective effects in many diseases [15, 23]. At present, there are few studies of TA on DN. In this research, we sought to investigate the effect of TA on DN and its potential mechanism. Our results revealed that TA exhibited crucial protective effect on renal function, alleviated histopathological changes, mitigated oxidative stress, ameliorated the inflammatory response, and reduced podocyte damage and fibrosis.

In this study, the therapeutic effects of TA in DN were detected in db/db mice. Our results showed that TA improved renal function by decreasing the levels of right kidney weight/body weight ratio, BUN, Scr, and urine albumin. Moreover, histological examination corroborated the therapeutic role of TA in lessening glomerular mesangial matrix expansion and collagen deposition in DN. In addition, TA administration potently decreased the expressions of COL-III and FN, which suggested that TA improved the deposition of extracellular matrix, inhibiting kidney fibrosis in DN.

Studies have shown that hyperglycemia, glucose metabolic upregulation, and reactive oxygen species (ROS) production could induce oxidative stress, which has been implicated in the pathogenesis of DN [24–26]. There are many mechanisms for the formation of ROS induced by hyperglycemia in diabetes, including the increase of advanced glycation end products, activation of protein kinase C, and the formation of superoxide anions through the mitochondrial electron transport chain [27]. Generally, ROS is necessary for normal metabolic conditions, whereas excessive ROS production in DN could impair cellular macromolecules including DNA, proteins, and lipids [28]. As one of the most important products of membrane lipid peroxidation, MDA is an important parameter reflecting the potential antioxidant capacity of the body. SOD and GSH are two vital antioxidants that protect cells from oxidative damage induced by ROS. CAT, a marker enzyme of peroxisomes, protects cells from the toxic effects of hydrogen peroxide [29]. In this study, our results showed that the decrease in GSH level and SOD and CAT activities and the increase in MDA content were remarkably reversed by TA treatment. We confirmed that TA administration increased the antioxidant capacity of mice and alleviated oxidative stress in DN. Similarly, Zhao et al. [19] also proved that TA had protective effects against oxidative stress injury induced by CCl_4_ in mice. Of note, Nrf2 is a critical transcription factor that regulates cellular redox homeostasis, which is essential for maintaining normal physiology. In oxidative stress conditions, activated Nrf2 translocates to the nucleus and binds to the antioxidant response elements to activate the downstream antioxidative enzymes HO-1 and NQO-1 [30]. Therefore, we suspected that Nrf2 signaling pathway participated in defending oxidative stress in DN. Subsequently, we found that after treatment with TA, the protein expression levels of Nrf2, HO-1, and NQO-1 were indeed dramatically increased. Our results showed ample evidence that TA mitigated oxidative stress through activating Nrf2/HO-1 mediated signaling pathway.

Other than oxidative stress, inflammation has emerged as being an indubitable pathophysiological mechanism of DN [6]. Inflammatory cytokines, produced in glomeruli, endothelial cells, and mesangial cells, play a key role in the pathogenesis of DN [6, 31]. Proinflammatory cytokine levels might be an early marker of renal dysfunction in DN [31]. In this study, we detected the expression levels of TNF-*α*, IL-1*β*, and IL-6, which indicated that TA inhibited the expression of inflammatory cytokines, thus playing a protective role in defending the inflammatory response in DN. Subsequently, we found that the potential mechanism of TA's anti-inflammatory effect was attributed to the inhibition of NF-*κ*B signaling pathway. Multiple studies have shown that NF-*κ*B, an important transcription factor, regulates the inflammatory response via mediating the expression of inflammatory cytokines [10, 32]. Our research demonstrated that TA administration strongly ameliorated renal inflammatory damage via inhibiting NF-*κ*B signaling pathway.

Podocyte damage occurs in the early stage of DN, which causes proteinuria and accelerates the development of DN [33]. Nephrin and podocin are two main podocyte cytoskeleton proteins, which jointly maintain the normal morphology and function of podocytes, thus maintaining the normal filtration barrier function of the glomerulus [34]. Therefore, the stability of nephrin and podocin proteins is vital to renal function. In this study, we unveiled that the elevated expression of nephrin and podocin proteins in DN was revised by TA treatment. Our results proved that TA exhibited a protective effect on podocyte damage in DN.

## 5. Conclusion

Our research provided potently evidences that TA exhibited renal protective effect by ameliorating oxidative stress and inflammation via Nrf2/HO-1 and NF-*κ*B signaling pathways. On account of the advantageous effect of TA on DN, it is plausible that *Inonotus obliquus* may be a new therapeutic strategy for the treatment of DN.

## Data Availability

The data that support the findings of this study are available in the article.
